# Expanding the Scope of PFAS Research to Include Adipose Tissues

**DOI:** 10.1021/acs.est.6c01695

**Published:** 2026-08-31

**Authors:** Xiaodi Shi, Matthew MacLeod, Amila O. De Silva, Karl J. Jobst, Mélanie Z. Lauria, Seth R. Newton, Jane Prihodova, Anna M. Roos, Anne L. Soerensen, Elsie M. Sunderland, Jonathan P. Benskin, Satoshi Endo

**Affiliations:** † Department of Environmental Science, 7675 Stockholm University, 10691 Stockholm, Sweden; ‡ Aquatic Contaminants Research Division, Environment and Climate Change Canada, Burlington, Ontario L7S 1A1, Canada; § Department of Chemistry, Memorial University of Newfoundland, St. John’s, Newfoundland and Labrador A1C 5S7, Canada; ∥ Department of Environmental Chemistry, Eawag, 8600 Dübendorf, Switzerland; ⊥ U.S. Environmental Protection Agency Office of Research and Development, Washington, D.C. 20460, United States; # Department of Environmental Monitoring and Research, 42763 Swedish Museum of Natural History, 10405 Stockholm, Sweden; 7 Harvard John A. Paulson School of Engineering & Applied Sciences, 1812 Harvard University, Cambridge, Massachusetts 02138, United States; 8 Department of Earth and Planetary Sciences, 1812 Harvard University, Cambridge, Massachusetts 02138, United States; 9 Health and Environmental Risk Division, National Institute for Environmental Studies (NIES), 305-8506 Tsukuba, Ibaraki, Japan

**Keywords:** Per- and polyfluoroalkyl substances (PFAS), lipid storage, partitioning behavior, exposure assessments

## Abstract

The occurrence and burden of per- and polyfluoroalkyl substances (PFAS) in adipose tissue of humans and wildlife represent an underexamined dimension of PFAS exposure. While most PFAS research has focused on ionic substances that accumulate in tissues such as liver and blood, there is growing evidence that a distinct and diverse suite of neutral PFAS bioaccumulate in adipose tissue. This Perspective synthesizes recent data illustrating the substantial contribution of some adipose-associated PFAS to overall body burdens, applies quantum chemical modeling to exemplify additional PFAS likely to partition into adipose tissue, and examines analytical blindspots that have limited their detection to date. We show that certain neutral PFAS possess partitioning properties comparable to legacy persistent organic pollutants, which, provided that they are sufficiently persistent, can lead to bioaccumulation in adipose tissue. We further outline the analytical challenge of extracting, detecting, and quantifying neutral PFAS in matrices rich in storage lipids and discuss the implications for exposure assessment, toxicology, and environmental surveillance. Finally, we identify key research needs and call for expanded monitoring strategies that capture the full chemical space of PFAS bioaccumulation.

## Graphical abstract

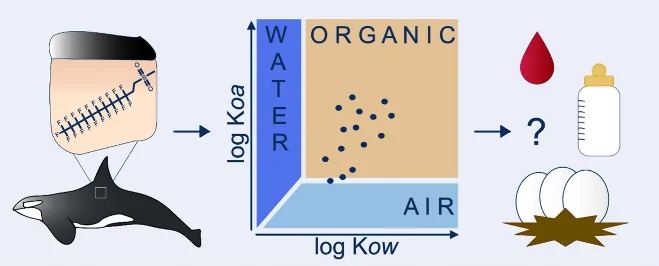

## Introduction

Owing to their extensive use in modern society and environmental persistence, per- and polyfluoroalkyl substances (PFAS) are globally distributed in the environment. Over seven million candidate structures in PubChem satisfy the OECD definition of PFAS, meaning they possess at least one CF_2_ (without a substituent hydrogen, chlorine, bromine, or iodine atom) or a CF_3_ group. The OECD PFAS definition encompasses a broad chemical space, ranging from hydrophilic, low-molecular-weight ions to hydrophobic, macromolecular polymers.^1^

The most widely studied PFAS, perfluoroalkyl acids (PFAAs), are strong acids and fully ionic at physiological pH, and strongly bioaccumulate in phospholipid- and protein-rich tissues such as liver and blood. These matrices have traditionally been the primary focus for identifying novel bioaccumulative PFAS. Adipose tissue, on the other hand, has been largely overlooked for PFAS, despite being the tissue where the majority of legacy persistent organic pollutants (POPs) bioaccumulate. A Web of Science search of abstract keywords illustrates this imbalance: “PFAS” and “adipose/storage lipids” yields only 19 results, compared to 1159 for “PFAS” and “liver/blood” (accessed 2025-12-20).

Adipose tissue is composed mainly of neutral storage lipids (e.g., triacylglycerols), and its percentage of bodyweight varies widely within human and wildlife populations. One study of the US National Health and Nutrition Examination Survey (NHANES) data sets found mean body fat percentages of 28% for men and 40% for women and defined “overweight” as 25% and 36% for men and women, respectively, and “obese” as 30% and 42%, respectively.^2^ In certain marine mammals adipose tissue can comprise up to 50% of total body mass.^3^ Neglecting adipose tissue therefore may lead to underestimation of overall PFAS body burdens in humans and wildlife. In this Perspective, our three main objectives are to (1) highlight a diverse suite of novel neutral PFAS detected in adipose tissue and environmental organic phases, (2) apply quantum chemical modeling to evaluate their tendency to partition to storage lipids, and (3) identify current analytical blindspots which hamper comprehensive exposure assessments of PFAS.

## Empirical Evidence for Accumulation of PFAS in Adipose Tissue

### (*N*-alkyl) Perfluoroalkane Sulfonamides ((P)­FASAs)

(P)­FASAs are semirecalcitrant neutral and/or weakly acidic precursors of PFAAs.^4^ They are widely detectable in the environment, and can be more bioaccumulative and toxic than PFAAs with the same number of perfluoroalkyl carbons.^5–7^ Phase-out initiatives combined with (a)­biotic transformation over time may be acting to reduce exposure to (P)­FASAs for humans and wildlife. However, an important exception appears to be in cetaceans, which have long lifetimes and lack key enzymes required for biotransforming certain PFAA-precursors;^8,9^ consequently, cetaceans face high (P)­FASA accumulation.^9–11^

Evaluations of (P)­FASA tissue distribution in North Atlantic pilot whales (*Globicephala melas*) and Greenland killer whales (*Orcinus orca*) have reported orders of magnitude lower concentrations in blubber than in liver.^10,12^ However, even low contaminant concentrations in blubber may significantly influence whole-body burdens in marine mammals, given the disproportionately large mass of adipose tissue (∼20% of total body mass in North Atlantic pilot whales and ∼35% in Greenland killer whales),^13,14^ compared with liver (∼0.8% and ∼3.5%, respectively).^14,15^ Indeed, under the assumption of fixed tissue mass fractions, the estimated perfluorooctane sulfonamide (FOSA) burden in blubber in these studies is 0.9–2.6 times that in liver, while the perfluorooctanesulfonic acid (PFOS) burden in blubber is estimated to be 0.3–1 times that in liver (Figure 1). Despite variability associated with individual body states, this comparable and even higher burden of FOSA in blubber, relative to liver, is considerable. Additionally, the larger proportion of FOSA in blubber, compared to PFOS, points to a higher affinity of (P)­FASA for adipose tissue compared to ionic PFAS.

**Figure 1:**
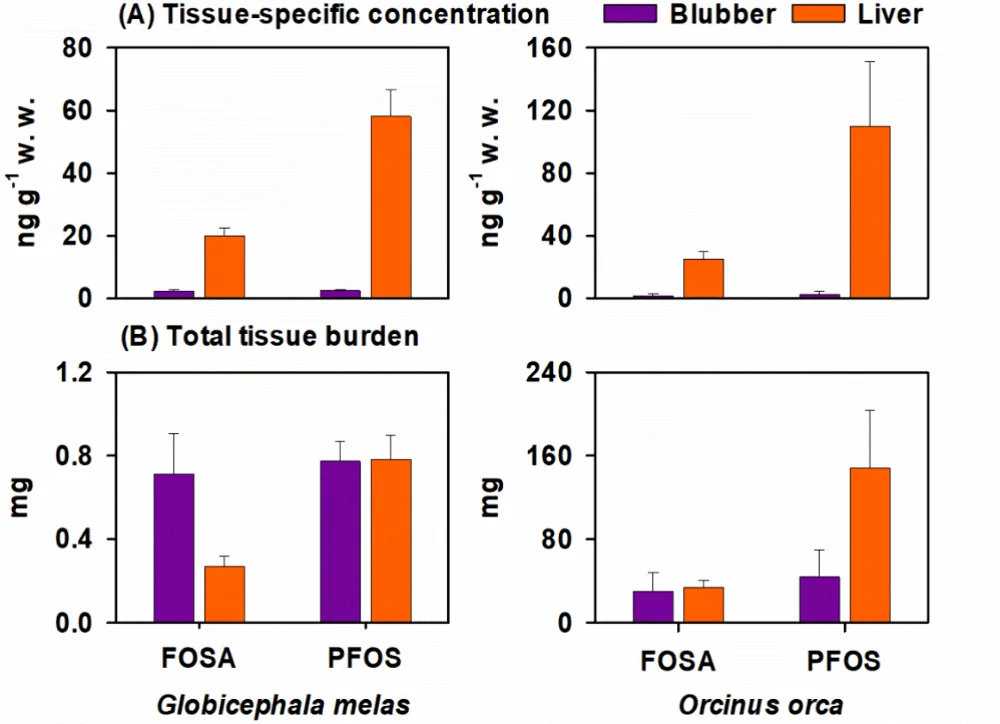
(A) Tissue-specific concentrations (wet weight); and (B) total tissue burdens of perfluorooctane sulfonamide (FOSA) and perfluorooctanesulfonic acid (PFOS) in North Atlantic pilot whales (*Globicephala melas*) and Greenland killer whales (*Orcinus orca*). The boxes and whiskers represent median and the third quartile for *Globicephala melas*, and average plus standard deviation for *Orcinus orca*. We assume that the whole-body mass of *Globicephala melas* is 1.55 t (20% blubber and 0.8% liver)^13,15^ and 45 t (35% blubber and 3.5% liver)^14^ for *Orcinus orca*. Tissue-specific concentrations are sourced from Dassuncao et al. 2019 and Schultes et al. 2020.^10,12^

FOSA and *N*-methyl perfluorooctane sulfonamidoacetic acid (MeFOSAA) have also been detected in human milk from Sweden and China, along with numerous PFAAs and alternatives.^16–19^ Notably, MeFOSAA displayed a higher transfer efficiency from maternal plasma to breast milk (6.75%) compared with PFAAs (0.20–2.08%),^19^ reflecting its significantly lower acidity (i.e., higher p*K*_a_) relative to fully ionic PFAAs. Measurements of paired whole blood, serum, and plasma samples from Norway and the US have also revealed that FOSA concentrations in whole blood were orders of magnitude higher than those in serum and plasma, while concentrations of PFOS and perfluorooctanoic acid in whole blood were about 0.5 times.^20,21^ These results highlight the need to investigate the distribution of neutral and partially ionizable PFAS among blood matrices as well as between blood and other organs.

### Fluorotelomer Sulfones

Following the finding of high unidentified extractable organic fluorine (EOF) in the blubber of a Greenland killer whale,^12^ a suite of fluorotelomer sulfones (FT-sulfones; i.e., 12:2 and 10:2 fluorotelomer methylsulfone, 12:2 and 10:2 fluorotelomer monochloromethylsulfone, and bis­(6:2 fluorotelomer) sulfone) were confirmed for the first time in wildlife. FT-sulfones were not detected in the liver of the same animals, and the fluorine mass balance was already closed by targeted PFAS, indicating a negligible accumulation in livers.^14^ These emerging compounds explained 34–75% of blubber EOF, and their total mass burden in blubber is estimated to be over twice that of previously quantified PFAS in liver. Detection of FT-sulfones in sediment from the Baltic Sea, the Arctic, a Norwegian lake and Chesapeake Bay (U.S.), and in wastewater treatment plant influent, effluent and river water from Spain, suggests that the occurrence of these substances is widespread.^22,23^ Their source(s) remain unclear, but possibilities include biotransformation of alkyl thiol-containing fluorotelomers, which are commonly used in aqueous film-forming foam and food packaging.^24,25^ For instance, formation of sulfone metabolites from a thiol precursor has been observed in marine mammals and soil microbes under aerobic conditions.^25,26^

### Fluorinated Liquid Crystal Monomers (LCMs)

Fluorinated LCMs are extensively applied in display devices.^27^ These substances typically possess a diphenyl or dicyclohexyl backbone with a few fluorine atoms on their aromatic ring/terminal functional group. In total, 578 fluorinated LCMs have been identified from articles and patents, and some meet the definition of PFAS.^27,28^ Due to their resemblance to some legacy POPs, LCMs are also the subject of increasing scrutiny as an emerging group of potentially persistent, bioaccumulative, and toxic substances. A total of 13 LCMs that satisfy the OECD definition of PFAS, including 4-(difluoro­(3,4,5-trifluorophenoxy)­methyl)-4′-ethyl-3,5-difluoro-1,1′-biphenyl (DTMDEB; C_21_H_13_F_7_O) and 2′,3,5-trifluoro-4″-(trans-4-propylcyclohexyl)-4-(trifluoromethoxy)-1,1′,4′,1″-terphenyl (TPrTT; C_28_H_26_F_6_O), have been detected in human samples (i.e., serum and milk).^29–31^ The propensity of fluorinated LCMs for adipose was further verified via *in vivo* exposure to mice and zebrafish.^32,33^

## Emerging Neutral PFAS in Abiotic Environmental Organic Phases

The small number of PFAS for which there is evidence of accumulation in adipose tissue as described in the previous section are mostly neutral or weak acids with high p*K*_a_, so a significant fraction remains neutral at physiological pH. A much wider suite of neutral PFAS have been detected in abiotic organic phases, and may be relevant for accumulation in storage lipids. For example, chlorofluoro phthalimides have similar structures to a known persistent and bioaccumulative flame retardant (i.e., CAS: 68815-72-5).^34^ These substances were tentatively identified for the first time in house dust by Zhang et al. (2019) and Newton et al. (2020),^35,36^ and then confirmed recently using authentic standards by MacNeil et al. (2022).^37^ The same study also detected chlorofluoroalkanes (some structural isomers of which contain −CF_2_/CF_3_ group(s) and meet PFAS under the OECD definition), which are used in a variety of applications from lubricants to nonstick coatings.^37,38^ None of these substances were quantified, but their occurrence in house dust indicates human exposure.^39^

Since sediment is a typical sink of hydrophobic contaminants, accumulation in sediment indicates relevance for potential lipid storage. Emerging neutral PFAS detected in sediment include fluorotelomer thiols and disulfides, as well as fluorinated methylsiloxanes. Fluorotelomer thiols and disulfides were identified in sediment impacted by a paper production plant in Norway at levels three times higher than the sum concentration of fluorotelomer sulfonates in the same lake.^22,25^ Fluorinated methylsiloxanes, including tris­(trifluoropropyl)­trimethylcyclotrisiloxane and tetrakis­(trifluoropropyl)­tetramethylcyclotetrasiloxane (also known as D3F and D4F), were reported for the first time as environmental contaminants by McLachlan et al. (2014) in sediments and municipal wastewater treatment plant sludge from Sweden, and Norwegian lake sediment.^40^ Subsequently, orders of magnitude higher concentrations were found in sediment near a manufacturing plant, sludge, and biosolid-amended soil in China.^41–43^

## Predicting Partitioning Properties of Neutral PFAS

Equilibrium partition ratios predicted using polyparameter linear free energy relationship (PP-LFER) models and the quantum chemical-based COSMO*therm* model indicate that (P)­FASAs and their analogues tend to distribute to the organic phase in a generic environment (air, water, and organic phase with a volume ratio of 10^6^:10^3^:1),^44–46^ which agrees well with their occurrence in adipose tissue.^10,12^ Here we expand the scope of COSMO*therm* predictions with new calculations for an additional 36 neutral fluorinated substances (Table 1), using methods identical to those described in Endo (2023).^46^ The selected compounds include those described in the previous sections and identified from patents. They cover a broad range of structures, including long-chain PFAS with diverse neutral (or partially neutral) head groups, LCMs with a few fluorine atoms, fluoro/chlorofluoroalkanes, and fluorinated organosilicones.

**Table 1:**
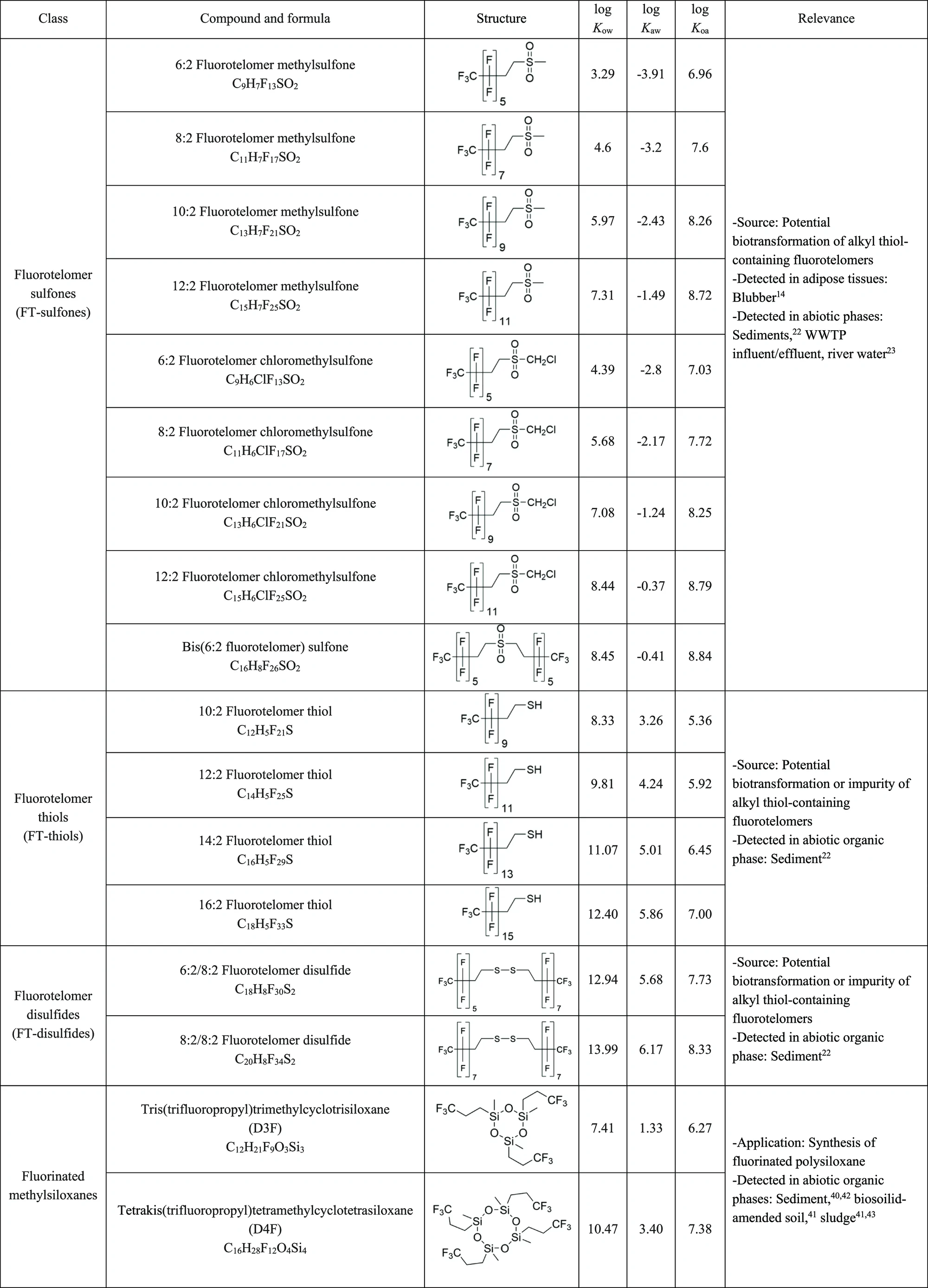

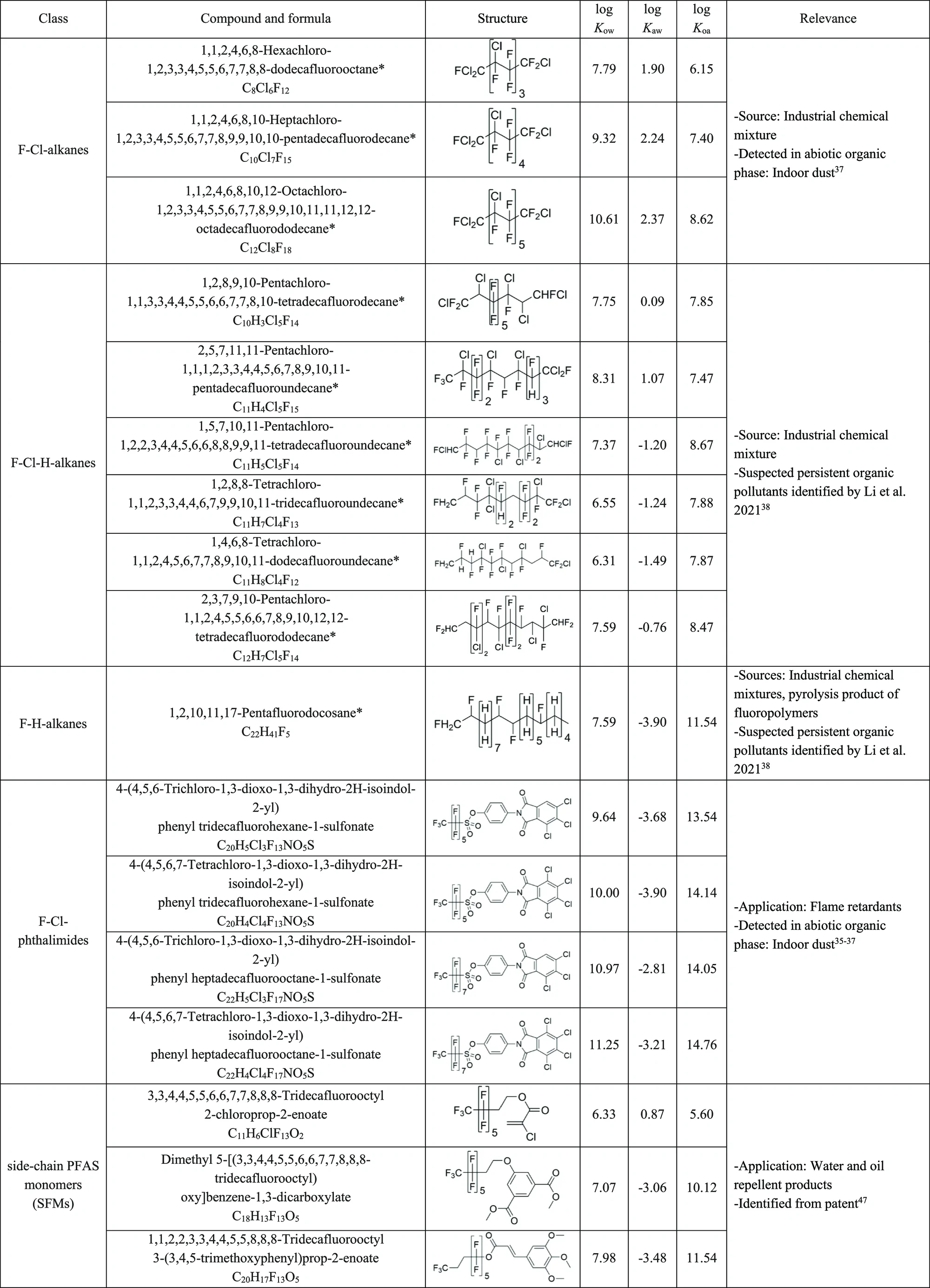

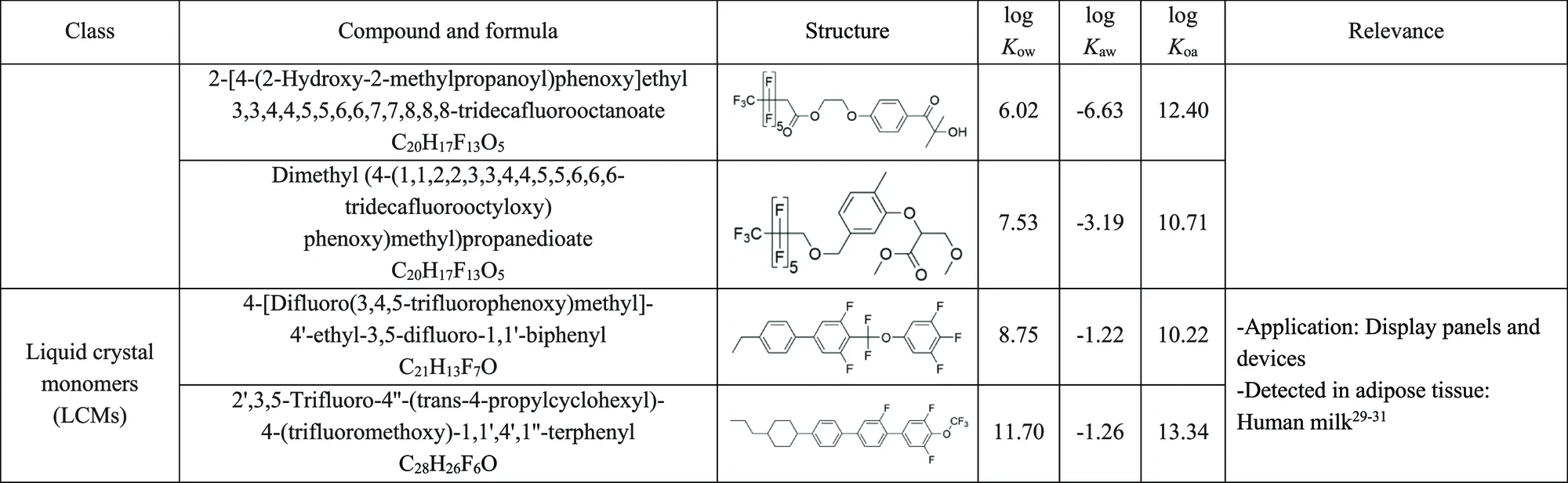
Equilibrium Partition Ratios for Neutral PFAS Recently Detected in Lipid-Rich Tissue, and/or Abiotic Organic Phases, Highlighted in Literature, or Described in Patents^a^

a *An arbitrary structure of all possible isomers, some of which meet the OECD definition of PFAS.

The predicted octanol-water, octanol-air, and air-water equilibrium partition ratios (*K*_ow_, *K*_oa_, and *K*_aw_, respectively) for these substances are provided in Table 1 and are plotted together with the properties of legacy POPs in Figure 2. The equilibrium mass in the organic phase of a generic environment is over 50% for most substances included here, indicating their high affinity for organic phases compared to air and water. In addition to previously predicted (P)­FASAs and *N*-alkyl perfluoroalkane sulfonamido ethanols (FASEs), the current results show that some side-chain PFAS monomers, fluorinated LCMs, chlorofluoroalkanes, and FT-sulfones display partition properties consistent with legacy POPs, e.g., polychlorinated biphenyls (PCBs), hexachlorobenzene (HCB), dichlorodiphenyltrichloroethane (DDT), and hexachlorocyclohexanes (HCHs; Figure 2). Among these compounds, chlorofluoroalkanes were previously predicted to be potential POPs based on density functional theory.^38^ These results show that certain neutral PFAS may bioaccumulate into adipose tissue through conventional food-web bioaccumulation mechanisms, provided that they are sufficiently persistent. Understanding of tissue partitioning and relative chemical solubility in different media is a sufficient theoretical construct for predicting bioaccumulation of many PFAS.^48,49^

**Figure 2:**
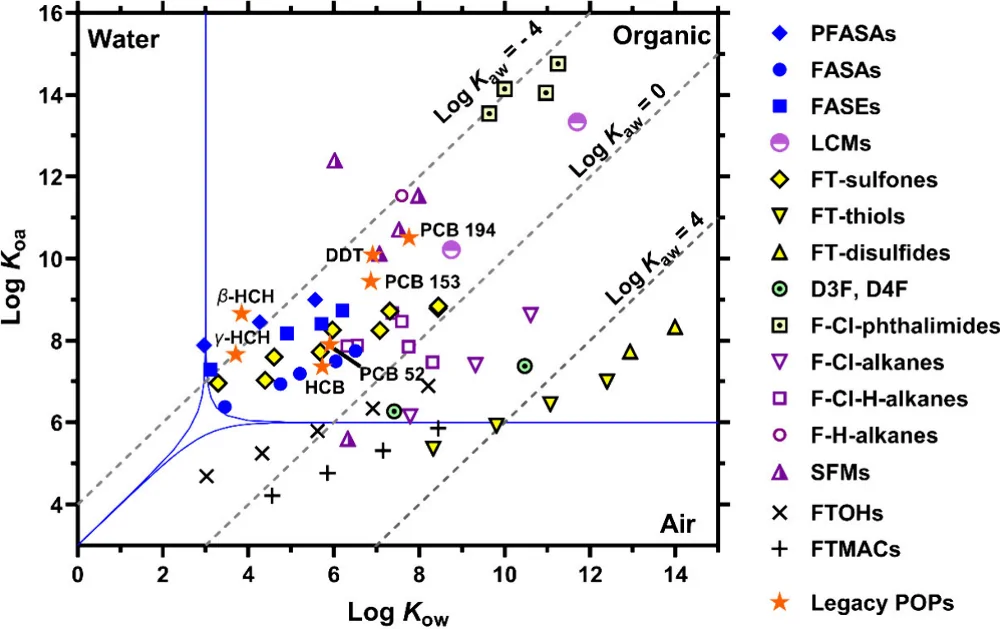
Data on chemical partitioning of novel neutral PFAS and selected legacy POPs. Partition ratios for sulfonamides, n:2 FTOHs (*n* = 4, 6, 8, 10, and 12), and n:2 FTMACs (*n* = 4, 6, 8, and 10) were predicted using polyparameter-linear free energy relationships (PP-LFERs) as reported in Endo 2023,^46^ while data for novel PFAS were calculated using COSMO*therm*. Please see Table 1 for identities and partition ratios for novel PFAS. Data for legacy POPs are experimental and from Baskaran et al. 2021, Xiao et al. 2004, Li et al. 2003, and EPISuite v. 4.1 (various sources).^50–53^ Solid lines indicate 50% by mass of the chemicals in the respective phases when they are distributed, at equilibrium, in a generic environment consisting of air, water, and organic phase with a volume ratio of 10^6^:10^3^:1, respectively. Dashed lines represent log *K*_aw_ values of −4, 0, and 4. Acronyms are as follows: β/γ-hexachlorocyclohexane (β/γ-HCH), hexachlorobenzene (HCB), polychlorinated biphenyl (PCB), and dichlorodiphenyltrichloroethane (DDT), perfluoroalkane sulfonamide (PFASA), *N*-alkyl perfluoroalkane sulfonamides (FASA), perfluoroalkane sulfonamido ethanol (FASE), fluorotelomer alcohol (FTOH), and fluorotelomer methacrylate (FTMAC). Acronyms for novel PFAS can be found in Table 1.

## Thermodynamic Controls on Bioaccumulation of Neutral PFAS in Adipose Tissue

Replacing hydrogen with fluorine in organic chemicals increases their molar volume. Molecules with a large molar volume require large cavities in any absorbing phase, which is energetically costly in water. High fluorination thus contributes to increased hydrophobicity and higher *K*_ow_ compared to nonfluorinated analogues,^54^ and neutral PFAS with long perfluoroalkyl chains generally have a strong tendency to partition from water to fat tissues. Nevertheless, PFAS have relatively low van der Waals interaction potential for their size, resulting in higher volatility from any condensed phase than nonfluorinated analogues.^54,55^ Indeed, frequently monitored neutral PFAS such as fluorotelomer alcohols (FTOHs) and fluorotelomer methacrylates (FTMACs) have low *K*_oa_ and high *K*_aw_ (Figure 2), readily volatilize into the atmosphere, and have not been reported to bioaccumulate in ecosystems. However, neutral long-chain PFAS containing highly polar functional groups (e.g., FT-sulfones), and large hydrocarbon-based organic molecules with just a single trifluoromethyl moiety (e.g., fluorinated LCMs), have sufficient affinity for condensed phases relative to the gas phase, to be bioaccumulative and found in fat tissues, like legacy POPs (Figure 2).

## Where and How to Search for PFAS That Bioaccumulate in Adipose Tissue

Empirical evidence and quantum chemical predictions offer examples of PFAS which accumulate in adipose. However, given the diversity and countless structures of PFAS, further comprehensive exposure assessments are required. Fish muscle and liver tissues remain the primary matrices for monitoring PFAS in wildlife, whereas eggs, adipose tissue, blood, and milk are analyzed less frequently. PFAS extraction procedures typically employ polar solvents which, while effective for PFAAs, have been shown to be less suitable for neutral substances.^56^ This may partially explain the large gaps between total fluorine and acetonitrile-extractable fluorine in eggs and blubber, albeit the importance of inorganic fluorine cannot be overlooked.^12,57^ In comparison, extraction of fluorinated LCMs from human milk and food samples has involved hexane mixtures followed by Florisil cleanup.^29,30,58^ These methods, and those originally developed for legacy POPs, may be adaptable for screening a broader range of neutral PFAS. To advance wildlife biomonitoring, future PFAS surveillance should expand to lipid-rich tissues and biofluids (e.g., milk, adipose tissue, whole blood, eggs) and incorporate updated preparation methods optimized for these matrices.

Instrumental analysis of novel PFAS has predominantly relied on liquid chromatography-electrospray ionization-high resolution mass spectrometry (LC-ESI-HRMS), a technique well suited for ionizable substances. However, recent amenability modeling suggests that LC-ESI methods may be applicable to fewer than 10% of known PFAS structures, based on evaluation of approximately 12,000 compounds.^59^ Alternative ionization methods such as atmospheric pressure chemical ionization and atmospheric pressure photoionization offer a possible improvement in amenability of LC-based approaches for neutral PFAS.^60,61^ Alternatively, coverage of this chemical space can be expanded to include neutral PFAS through complementary gas chromatography (GC) methods. In support of this, an extensive GC-HRMS PFAS library has recently been established covering over 1,900 electron ionization and positive chemical ionization spectra (predicted and experimental) for nontarget and suspect screening.^62^ For *de novo* structural elucidation of neutral PFAS, however, soft ionization approaches (e.g., atmospheric pressure chemical ionization and atmospheric pressure photoionization) are necessary to preserve molecular ions. These approaches have been applied for identification of many of the novel substances described here,^14,22,37^ along with quantification of FTOHs, FASEs, FASAs, and fluorinated LCMs.^29,58,63^

Alternative approaches that are free of selectivity of chromatographic system, such as ambient mass spectrometry coupled to plasma-based ionization (e.g., direct analysis in real time [DART] and laser-induced plasma ionization) may also hold potential for neutral PFAS.^61^ Plasma-based chemical ionization techniques are driven by ion–molecule reactions and therefore may be suitable for ionizing both nonpolar and polar molecules.^64,65^ Such techniques also enable rapid, *in situ*, and real time analysis. A similarly broad coverage can also be achieved from ^19^F nuclear magnetic resonance (^19^F-NMR) spectroscopy, an approach that offers both quantitative and structural information. Current ^19^F-NMR spectral databases collectively contain 647 fluorinated compounds, 431 of which are PFAS, including PFAAs, fluorotelomers, and agricultural and pharmaceutical chemicals with an aromatic trifluoromethyl group.^66,67^ This coverage can be further extended through computational chemistry and machine-learning prediction.^67,68^ However, further application of ^19^F NMR still faces difficulties in sensitivity and spectral overlap.^66^

Lastly, computational chemistry tools offer a valuable approach for prioritizing PFAS that tend to partition into adipose tissue from existing inventories. Quantum chemistry based COSMO*therm* has been shown to reliably predict the partitioning behavior of neutral PFAS based solely on molecular structure.^46,47^ Alternative strategies include empirical models such as PP-LFERs, quantitative structure property relationships, and emerging machine learning techniques. However, reliable experimental partition data for neutral PFAS remain limited,^46,69^ constraining model training and, consequently, limiting predictive accuracy for a broad spectrum of PFAS.

## Implications for Future Research

Empirical measurements have identified a small set of neutral PFAS that bioaccumulate in adipose tissue, and model predictions (some reported here for the first time) indicate that many other such PFAS likely exist. In the absence of systematic investigation into these substances, total PFAS exposure burdens are likely underestimated. Indeed, recent nontarget screening of marine mammal blubber flagged >300 potential unknown organofluorines.^14^ PFAS occurrence in adipose may have unique toxicological implications. In humans, the rapid rise in the use of glucagon-like peptide-1 (GLP-1) receptor agonists may represent an unexplored pathway for contaminant mobilization as adipose stores are rapidly depleted. Comparable processes occur naturally in Arctic wildlife during seasons of high energetic demand driven by fluctuations in temperature, food availability, and the energetic costs of migration, reproduction, and molting.^70–72^ Under these conditions, mobilization of fat reserves can cause contaminants in adipose tissue to be released into the bloodstream, potentially reaching vital organs and becoming further concentrated in the remaining fat stores.^73,74^ We call for increased research on this novel class of PFAS, including analytical techniques, sources, tissue distribution, toxicological effects, persistence, and partitioning behavior, to improve the overall assessment of risks of PFAS.
